# Suppressing Kaposi’s Sarcoma-Associated Herpesvirus Lytic Gene Expression and Replication by RNase P Ribozyme

**DOI:** 10.3390/molecules28083619

**Published:** 2023-04-21

**Authors:** Yujun Liu, Yuan-Chuan Chen, Bin Yan, Fenyong Liu

**Affiliations:** 1 School of Public Health, University of California, Berkeley, CA 94720, USA; 2 Program in Comparative Biochemistry, University of California, Berkeley, CA 94720, USA

**Keywords:** Kaposi sarcoma-associated herpesvirus, herpesvirus, antiviral, gene therapy, RNase P, ribozyme, catalytic RNA

## Abstract

Kaposi’s sarcoma, an AIDS-defining illness, is caused by Kaposi’s sarcoma-associated herpesvirus (KSHV), an oncogenic virus. In this study, we engineered ribozymes derived from ribonuclease P (RNase P) catalytic RNA with targeting against the mRNA encoding KSHV immediate early replication and transcription activator (RTA), which is vital for KSHV gene expression. The functional ribozyme F-RTA efficiently sliced the RTA mRNA sequence in vitro. In cells, KSHV production was suppressed with ribozyme F-RTA expression by 250-fold, and RTA expression was suppressed by 92–94%. In contrast, expression of control ribozymes hardly affected RTA expression or viral production. Further studies revealed both overall KSHV early and late gene expression and viral growth decreased because of F-RTA-facilitated suppression of RTA expression. Our results indicate the first instance of RNase P ribozymes having potential for use in anti-KSHV therapy.

## 1. Introduction

Kaposi’s sarcoma-associated herpesvirus (KSHV) (designated as human herpesvirus 8, HHV-8) is a member of the human herpesvirus family, which also includes herpes simplex viruses 1 and 2 (HSV-1 and 2), Varicella Zoster virus (VZV), human cytomegalovirus (HCMV), Epstein–Barr virus (EBV), as well as human herpesvirus 6 and 7 [1]. This virus instigates oncogenic manifestations such as primary effusion lymphoma (PEL) and Kaposi’s sarcoma (KS), which is among the leading neoplasms in AIDS patients [2,3]. The current dearth of available antiviral compounds or strategies for the treatment of KSHV-associated diseases necessitates ongoing development of unique and effective antiviral treatments against KSHV infection.

As with other herpesviruses, KSHV can result in both lytic and latent infections [2]. KSHV pathogenesis and the development of KS has been critically associated with the spontaneous start of the lytic cycle [4,5]. Successful therapy against KSHV infection requires effective viral lytic replication blockade.

**Figure 1:**
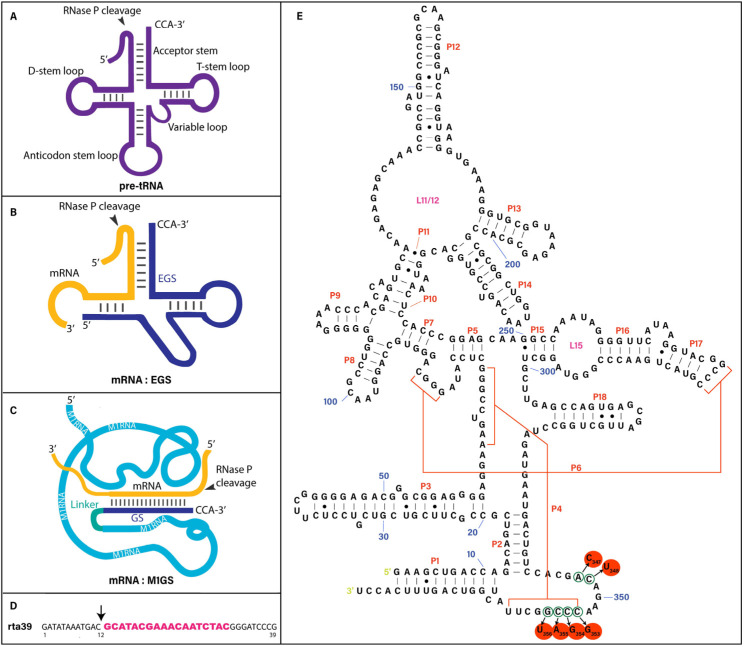
(**A**,**B**) Substrates for RNase P and M1 RNA. (**C**) Substrate for M1GS ribozyme. (**D**) rta39 substrate in this study. (**E**) The mutated positions in ribozyme C-RTA (circled and in red), compared to F-RTA, shown in the secondary structure of M1 RNA [13,14]. The cleavage site is marked by an arrow.

The scientific community has identified ribozymes as promising agents for silencing genes and inhibiting viral growth by creating cleavage in viral mRNA sequences [6,7,8]. Ribonuclease P (RNase P) is an enzyme responsible for slicing a 5′ leader sequence from a tRNA precursor (pre-tRNA) during tRNA maturation (Figure 1A) [9,10]. In *Escherichia coli*, RNase P is composed of a C5 protein subunit and a catalytic M1 RNA subunit [11]. In the absence of C5 protein, M1 RNA alone can efficiently cleave the tRNA precursor substrates under 100 mM MgCl_2_ [11]. The enzymatic activity of RNase P is highly active and efficient because this enzyme is responsible for the processing and maturation of more than 70 different species of tRNAs in *E. coli*. RNase P catalytic RNAs exhibited defined secondary structures (Figure 1E) and three-dimensional structures of active conformations [9,12]. For example, the highly conserved P4 helix and its surrounding sequences in M1 RNA are located near the active site and are required for its catalytic activity (Figure 1E). Due to these distinct biological properties, RNase P catalytic RNAs such as M1 RNA may represent a novel class of nucleic acid-based gene interference agents for gene-targeting applications.

RNase P and M1 RNA primarily recognize the overall three-dimensional structures of the substrates instead of their primary sequences (Figure 1A,B). Altman and colleagues previously established that RNase P and M1 ribozyme hydrolyze mRNA targets given the presence of an engineered external guide sequence (EGS) to hybridize with the mRNA, leading to a structure with resemblance to the portion of a tRNA substrate which includes the acceptor stem, the T-stem, the 3′ CCA sequence, and the 5′ leader sequence (Figure 1A–C) [15,16]. We can create a ribozyme known as M1GS RNA to interact with an mRNA target of interest using covalent linkage between the 3′ terminus of M1 RNA and a guide sequence (GS) (Figure 1C) [17,18]. M1GS ribozymes have previously shown the ability to hydrolyze human and viral mRNAs, and to decrease gene expression and growth in various human viruses such as HSV-1, HCMV, and human immunodeficiency virus (HIV-1) [19,20,21,22].

In this report, M1GS ribozymes were assembled to target KSHV replication and transcription activator (RTA) mRNA, a necessary component for viral infection [2]. RTA is an immediate-early gene encoding a transcription factor that activates the expression of most KSHV early and late genes [23,24,25,26]. We revealed the capability of the engineered RNase P ribozyme to cleave the RTA mRNA sequence in vitro, with decreased RTA expression detected in KSHV-infected human B cells. Furthermore, both viral early and late gene expression and virus production exhibited a substantial decrease in cells expressing the functional ribozyme. These results demonstrate that the engineered RNase P-based ribozyme can be utilized as a gene-silencing tool for the inhibition of KSHV gene expression and associated infection.

## 2. Results

### 2.1. In Vitro Cleavage of KSHV RTA mRNA by RNase P Ribozyme

To identify RTA mRNA regions that are potentially exposed to ribozyme binding, dimethyl sulphate (DMS) was used to treat BCBL-1 cells latently infected with KSHV. In vivo mapping with DMS [17,27,28] has been used to determine the unfolded and exposed regions of various mRNAs in cells and to assess the accessibility of these mRNA regions. At least nine different RTA mRNA regions were found to be exposed to DMS modification (Supplemental Table S1). A position 97 nucleotides downstream from the 5′ terminus of RTA mRNA exon 2 [26] became our choice for the cutting site of the M1GS ribozyme. This region was shown to be exposed and substantially modified by DMS. Furthermore, the site of cleavage is also in close proximity to the splicing junction and is likely exposed to ribozyme binding. Its flanking sequences also possess distinctive characteristics and components known to be important for substrate recognition by RNase P and M1 RNA [9]. For example, the cleavage site and its flanking sequence contain a guanosine immediately 3′ adjacent to the site of cleavage, and a uridine or cytosine immediately upstream of the site of cleavage (Figure 1D). M1 RNA and M1GS ribozyme have been shown to interact with these unique sequence components for optimal substrate recognition and efficient cleavage [6,9].

We engineered the functional and control inactive M1GS ribozymes, F-RTA and C-RTA, by covalently joining the 3′ end of the ribozyme M1 RNA and C102 RNA to an 18nt long guide sequence against the target RTA mRNA sequence, respectively (Figure 1C). M1 RNA was used to develop the inactive ribozyme mutant C102 RNA, with its P4 catalytic domain and surrounding regions containing various point mutations (i.e., A_347_C_348_ → C_347_U_348_, C_353_C_354_C_355_G_356_ → G_353_G_354_A_355_U_356_) (Figure 1E), and C102 is at minimum 1 × 10^5^-fold less active compared to the original M1 ribozyme [29]. The P4 helix and its surrounding sequences are highly conserved among RNase P catalytic RNAs and are important for RNase P ribozyme active conformation and catalysis (Figure 1E) [12,13,14]. Our results are consistent with previous observations that mutations at the P4 helix region abolished the cleavage activity of M1GS [9,29].

To determine the effect of an M1GS RNA that does not target RTA mRNA, we derived M1-TK, another control ribozyme, from M1 RNA. M1-TK ribozyme was constructed by linking to the 3′ terminus of M1 RNA with an 18-nucleotide-long guide sequence targeting the mRNA encoding the thymidine kinase (TK) of herpes simplex virus 1 (HSV-1). This ribozyme was previously shown to cleave the TK mRNA sequence efficiently in vitro and inhibit TK expression effectively in HSV-1 infected cells [17,30].

Upon introduction of F-RTA, we detected the slicing of RNA substrate rta-39 (Figure 1D), consisting of 39 nucleotides from the targeted RTA mRNA (Table 1). Due to point mutations in the catalytic center region of C-RTA (Figure 1E), this ribozyme was barely active in cleaving rta-39, as measured by the overall cleavage rate (k_cat_/K_m_)^s^ (Table 1). M1-TK was not active in cleaving rta-39 due to its incorrect targeting sequence. Similar results were also observed when F-RTA, C-RTA, and M1-TK were incubated with a 680-nucleotide-long RTA mRNA substrate. These observations imply that F-RTA is active in slicing the full-length RTA mRNA while C-RTA and M1-TK are not active.

To examine if the variation in binding affinities produced separate ribozyme cleavage efficiencies, the binding affinities of the ribozymes to substrate rta-39, measured as the dissociation constant (K_d_), were extrapolated with gel shift assays. The binding affinities of C-RTA were comparable to those of F-RTA (Table 1). Thus, C-RTA was used as an antisense effect control since it presented equivalent affinity to rta-39 as F-RTA but was inactive.

### 2.2. Ribozyme Expression in Human Cell Lines

We used the LXSN retroviral vector expression system to express F-RTA and C-RTA ribozymes under the small nuclear U6 RNA promoter in human cells [17,31]. LXSN vector has been previously used to express M1GS ribozyme in human cells [30,32]. We initially inserted the DNAs for F-RTA, C-RTA, and M1-TK ribozymes into the LXSN DNA at a location downstream of the U6 RNA promoter. LXSN-M1GS DNAs and empty LXSN vector without any ribozyme sequences were then transfected into human BCBL-1 cells latently infected with KSHV, and the cells containing the LXSN sequence were cloned.

**Figure 2:**
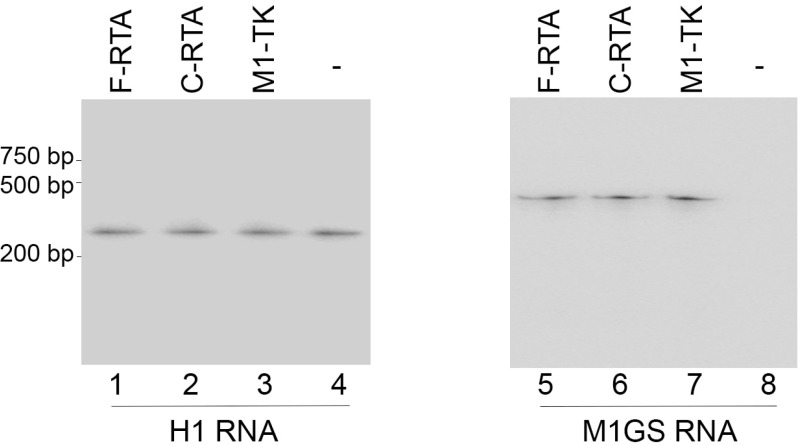
Northern blot analysis of cellular ribozyme expression. RNAs (30 µg) from BCBL-1 cells transfected with empty LXSN vector with no ribozyme sequences (-) and cells with different ribozymes (F-RTA, C-RTA, and M1-TK) were hybridized with probes against human H1 RNA (loading control) (lanes 1–4) and M1GS RNAs (lanes 5–8).

Northern blot experiments revealed ribozyme expression in different cell clones and showed that the level of F-RTA was similar to that of C-RTA in the cell clones examined (Figure 2, lanes 5–8). The level of human RNase P RNA, H1 RNA, served as an internal loading control (Figure 2, lanes 1–4). These experiments indicated that the cell lines had similar levels of F-RTA, C-RTA, and M1-TK ribozymes. Neither cell viability nor growth exhibited significant differences between the BCBL-1 cells transfected with empty LXSN vector without any ribozyme sequences and the cell lines containing LSXN-M1GS DNAs, for a span of three months. These results imply that ribozyme expression displayed little apparent cytotoxicity.

### 2.3. Suppression of RTA Expression by F-RTA

**Figure 3:**
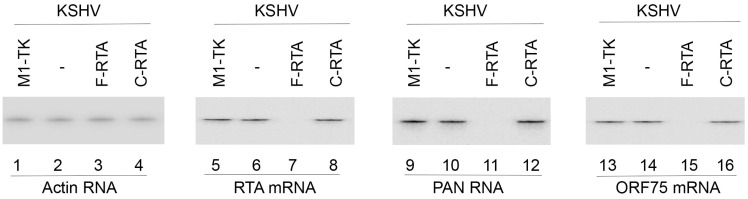
Expression of viral RNAs detected by Northern blot analysis. RNAs (40 µg) from BCBL-1 cells with no ribozyme sequences (-) and cells with different ribozymes (F-RTA, C-RTA, and M1-TK) were hybridized with probes for actin mRNA (lanes 1–4), RTA mRNA (lanes 5–8), PAN RNA (lanes 9–12), and ORF75 mRNA (lanes 13–16).

**Figure 4:**
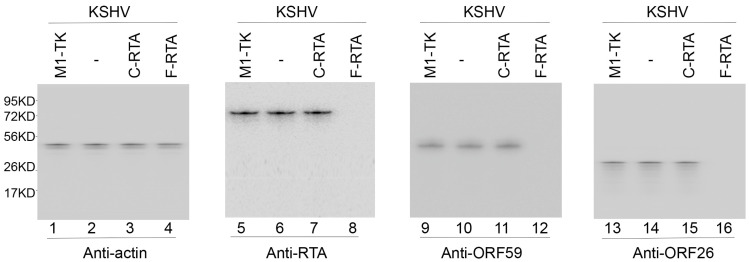
Expression of KSHV proteins detected by Western blot analysis. Proteins (35 µg) from BCBL-1 cells with no ribozyme sequences (-) and cells with different ribozymes (F-RTA, C-RTA, and M1-TK) were allowed to bind to antibodies specifically against human actin (loading control), RTA protein, ORF59 protein, and ORF26 protein.

To investigate the ribozyme efficacy in affecting RTA expression, cells were incubated in the presence of TPA to induce the KSHV lytic cycle and then harvested with total RNAs and proteins collected. We employed Northern and Western blot experiments to monitor the levels of RTA mRNA and protein, respectively (Figure 3, lanes 5–8, Figure 4, lanes 5–8), with β-actin expression (Figure 3, lanes 1–4, Figure 4, lanes 1–4) as the internal loading control. In cells expressing F-RTA, C-RTA, and M1-TK, RTA mRNA and protein expression decreased by about 92–94%, 5–7%, 0%, respectively (Table 2). From this, we infer the antisense effect to be the explanation for the low inhibitory outcome in cells expressing C-RTA, because C-RTA and F-RTA share the same guide sequence. These results suggest that F-RTA cleaved the RTA mRNA and was responsible for the observed RTA expression decrease in BCBL-1 cells.

### 2.4. Suppression of KSHV Infection by F-RTA

Previous studies have demonstrated that RTA functions as a major transcription activator responsible for KSHV reactivation from latent infection to lytic infection [2]. Specifically, RTA expression is required for induced expression of KSHV early (E) and late (L) genes [23,24,25,26]. Thus, KSHV early and late gene expressions would be suppressed following a reduction in RTA level in F-RTA-expressing cells. To verify if this is the case, ribozyme-expressing BCBL-1 cells were incubated with TPA, with total RNAs and proteins isolated from these cells at various periods after TPA treatment.

We monitored the levels of PAN RNA (an early RNA) and ORF75 (an early/late mRNA) (Figure 3, lanes 9–16) in addition to the protein levels of ORF26 (an early/late protein), ORF59 (an early protein) and K8.1 (a late protein) (Figure 4, lanes 9–16, Table 2). The levels of human actin mRNA and protein were used as internal loading controls. A decline of approximately 80–85% in their expressions was documented in the F-RTA-expressing cells (Table 2). The expression of viral mRNAs and proteins did not display meaningful changes in cells expressing C-RTA and M1-TK (Figure 3 and Figure 4, Table 2). Thus, the F-RTA-expressing cells exhibited an overall repression of viral early and late genes.

**Figure 5:**
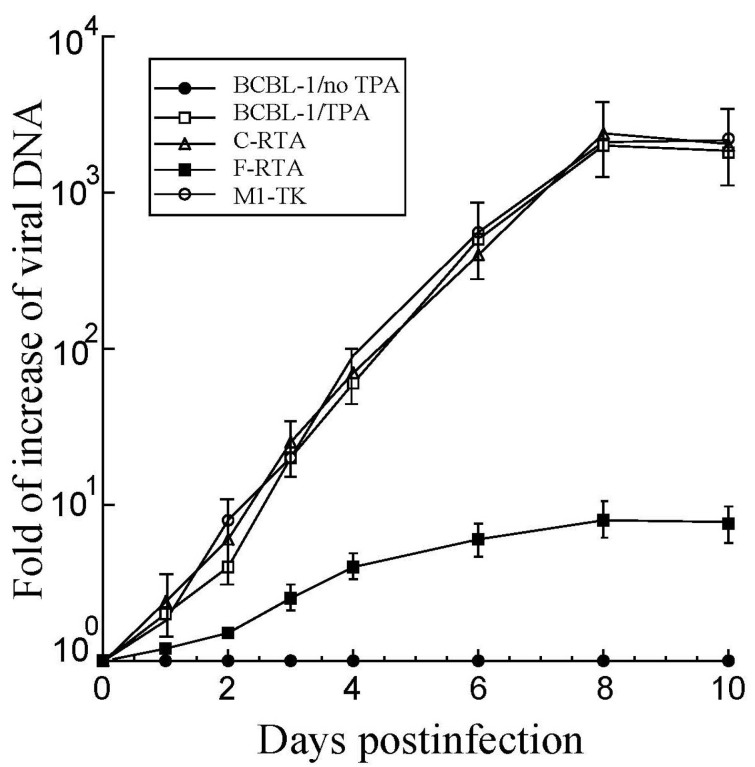
Levels of KSHV DNAs in supernatants of the cell cultures by qPCR. DNAs were purified from supernatants of cell cultures treated with or without TPA and collected at different time points. We employed qPCR to determine viral DNA levels, following procedures described in Materials and Methods. The values show the increase in virion DNA level in the samples, compared to that of virion DNA level in BCBL-1 cells with empty pLXSN vector in the absence of TPA (BCBL-1 no TPA). The averages of three independent experiments are shown and bars indicate the standard deviations.

Our next series of experiments focused on establishing if viral reproduction was impacted in cells expressing the designed ribozymes. Previous reports stated that performing viral plaque assays and measuring plaque-forming units is technically challenging for KSHV particles from BCBL-1 cells [33,34]. The level of viral DNA released into the media was assayed as an indicator of the level of viral virions. TPA was incubated with the ribozyme-expressing BCBL-1 cells. Cell culture supernatants were harvested during the time course post-TPA treatment, with subsequent treatment of DNase I. Extracellular virion DNA levels, represented by DNase-resistant viral DNA levels in the supernatants, were quantified with qPCR via primers amplifying the KSHV ORF25 sequence. Prior to TPA induction, the supernatants from the cultures of parental BCBL-1 cells or the cells transfected with empty LXSN vector without any ribozyme sequences hardly had any DNase I-resistant KSHV DNA (Figure 5). However, considerable quantities of KSHV DNA were detected in the supernatants with these cells upon TPA incubation, indicating robust KSHV intracellular reactivation and viral production (Figure 5).

**Figure 6:**
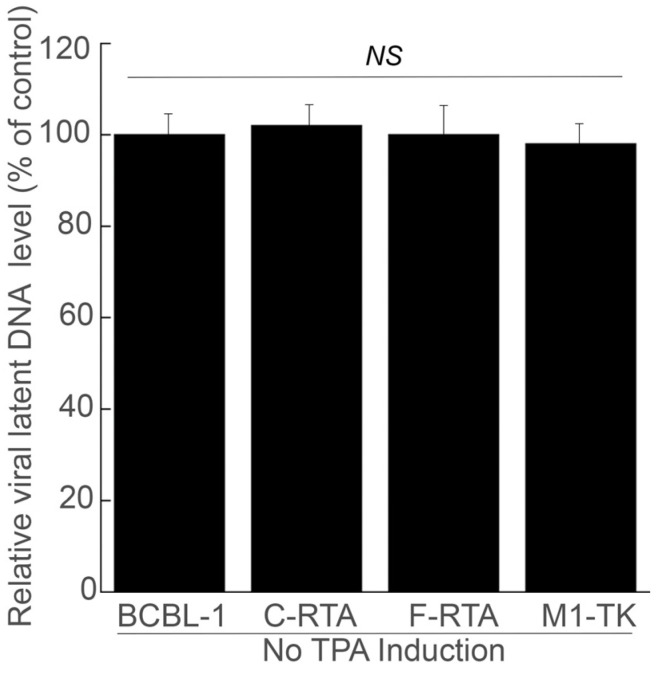
KSHV latent genome DNA levels in cells by qPCR. We harvested DNA samples from cells without TPA induction and performed qPCR assays. The values show the relative percentage of viral genome DNA levels in samples, as compared with the level of KSHV genome DNA in the BCBL-1 cells transfected with empty pLXSN vector and no ribozyme sequence (BCBL-1). The averages of three independent experiments are shown and bars indicate the standard deviations. NS, not significant.

A significant quantity of DNase-resistant viral DNA was identified in the supernatants of cell cultures without ribozymes, with C-RTA, or with M1-TK (Figure 5). By comparison, severely low amounts of DNase-resistant viral DNA were found in F-RTA cell-derived supernatants. Meanwhile, qPCR assays revealed comparable intracellular viral DNA levels in these cells prior to TPA treatment (Figure 6), indicating little difference in latent KSHV genome levels. The findings above imply that the diminished DNase-resistant viral DNA levels observed in F-RTA expressing cells are the result of F-RTA catalytic cleavage of RTA mRNA, rather than due to a reduced quantity of viral latent genome DNA. At 8 days after infection, F-RTA-expressing cells yielded an about 250-fold suppression in viral production (Figure 5). In cells with the control ribozymes C-RTA or M1-TK, the reduction in viral production was unremarkable (Figure 5).

## 3. Discussion

Ribozymes, such as those derived from M1 RNA, exhibit promise for inhibiting viral gene expression and growth because RNase P catalytic RNA appears to be among the most active RNA enzymes found in nature [6,8]. We report here the evidence of engineered RNase P ribozyme cleaving the KSHV RTA mRNA sequence in vitro and effectively suppressing RTA expression and KSHV production in human cells. In TPA-induced BCBL-1 cells, F-RTA expression suppressed RTA levels by 92–94% and virus production by about 250-fold while C-RTA and M1-TK expression did not significantly change RTA and virus production levels when compared to the BCBL-1 cells without any ribozyme expression. Ribozyme C-RTA, which shares the same guide sequence as F-RTA, did bind to the RTA mRNA sequence (i.e., rta-39) as well as F-RTA in vitro (measured as K_d_) but lacked catalytic activity (Table 1). M1-TK, which targets the HSV-1 TK mRNA, served as a control to examine the effect of an M1GS ribozyme with an incorrect guide sequence on RTA expression. Hence, the results reported here suggest the observed RTA expression reduction by F-RTA is largely caused by ribozyme-mediated cleavage of targeted RTA mRNA, rather than stemming from nonspecific effects of ribozymes such as the antisense effect of the guide sequence.

The cleavage of RTA mRNA induced by F-RTA seems to be the reason for the antiviral effect of RNase P ribozymes. Moreover, F-RTA-mediated targeting is specific. First, cells with these ribozymes had comparable growth and viability to cells transfected with empty LXSN vector without any ribozyme sequences for up to 3 months, indicating a lack of substantial cytotoxicity from ribozyme expression. The levels of human H1 RNA and actin mRNA in cells with or without ribozyme sequences had no significant disparity. Second, the antiviral effect of F-RTA is due to the suppression of RTA expression, since the expression of all investigated viral early and late genes (e.g., PAN RNA, ORF75, ORF59, ORF26, and K8.1) in the study preferentially decreased in cells expressing F-RTA, but those expressing C-RTA or M1-TK revealed no change (Figure 3 and Figure 4, Table 2). Furthermore, the extent of the suppression of RTA expression correlated with that of the suppression of viral early and late gene expression. Interestingly, there was no notable decline detected in the expression of other viral immediate early (IE) genes (e.g., K3 mRNA and ORF45) in cells expressing F-RTA, C-RTA, or M1-TK. Thus, the M1GS ribozyme acts selectively against its target mRNA.

Reactivation of the KSHV lytic cycle is critical to viral pathogenesis and development of KS [4,5]. The RTA protein is central to the KSHV reactivation process, due to its critical function of reactivating the KSHV genome from the latent state to lytic mode, and is necessary for viral early and late gene expression [2]. Hence, suppressing RTA expression by RNase P ribozyme may represent a novel methodology for effective anti-KSHV therapy.

Looking forward, it is important to demonstrate the feasibility of testing this approach in vivo. We need to confirm if M1GS RNA (e.g., F-RTA) is effective in suppressing viral lytic infection in primary cells known to be infected by KSHV in vivo, such as different populations of B cells [2,35,36]. Experiments will be carried out to determine if M1GS ribozyme can slice RTA mRNA and diminish viral RTA and other gene expression in these cells.

We will also need to address several potential challenges for using RNase P ribozymes for the treatment of KSHV infection in vivo. First, it will be necessary to improve the targeting activity of the ribozymes in vivo. The activities of M1GS RNA and M1 RNA in human cells have not been extensively studied. In the absence of C5 protein, M1 RNA (and M1GS RNA) can efficiently cleave their substrates only in the presence of high ionic strength (e.g., 100 mM MgCl_2_) [11,17]. M1 RNA activity can be enhanced by C5 protein as well as specific human RNase P protein subunits [9,37]. Recent studies indicated that hydrophobic-cationic peptides modulate various ribozyme activities by accretion and other effects, and Mg^++^ ions can have either stimulatory or inhibitory effects depending on their concentrations and the ribozymes examined [38,39]. Consistent with previous studies [9,37], these results highlight the important roles of Mg^++^ ions in RNase P ribozyme catalytic activity and suggest that M1GS ribozyme activity can be profoundly influenced by cellular environments with different ionic strengths and by different human proteins. Additional studies are needed to investigate the roles of Mg^++^ ions and human proteins in modulating RNase P ribozyme activity, especially in the context of normal physiological conditions and cellular environments.

Previously, we used in vitro selection procedures to produce M1GS ribozyme variants exhibiting improved catalytic activity to cleave target mRNA in vitro [30,40]. When expressed in human cells, these variants are more effective at inhibiting the expression of their target mRNAs as compared to ribozymes derived from the wildtype M1 RNA sequence. By carrying out the selection procedures in the presence of protein extracts from human cells, we can potentially generate ribozyme variants with better cleavage activity in human cells. It will be interesting to examine whether M1GS ribozymes derived from some of these variants are also more effective in inhibiting RTA expression and KSHV production than F-RTA.

Second, we need to develop effective delivery methods to better utilize RNase P-based ribozymes as anti-KHSV therapy. For example, efforts are needed to investigate how to deliver the ribozyme expression cassettes to cells latently infected with KSHV, and how to express M1GS ribozymes at a high level in these cells. Newly constructed viral vectors were shown to be successful in the delivery of therapeutic ribozyme expression cassettes in vivo [41,42]. These vectors can be used to deliver the M1GS expression cassettes in human cells such as B cells, which are known to be latently infected with KSHV [2]. In previous anti-HIV ribozyme clinical studies, a gene therapy approach was used and ribozymes were expressed via retroviral and lentiviral vectors in CD34+ hematopoietic stem cells, which differentiate into hematopoietic lineage cells including T and B cells [42,43,44,45]. These studies indicated that the gene-delivered ribozymes are safe and feasible for anti-HIV therapy [41]. In principle, the anti-KSHV ribozymes can be delivered and expressed in B cells for anti-KSHV therapy using similar approaches. We also need to address other potential challenges such as reducing immune toxicity and stimulation associated with ribozymes and mass-producing the ribozyme lentiviral vectors for clinical studies. Further investigations on these issues and addressing these challenges will facilitate the development of RNase P ribozymes for anti-KSHV applications.

## 4. Materials and Methods

### 4.1. Antibodies, Viruses, and Cells

We grew human PEL BCBL-1 cells containing KSHV and induced KSHV reactivation in these cells with tetradecanoyl phorbol acetate (TPA) (20 ng/mL) (Sigma, St. Louis, MO, USA), as noted previously [34,46]. Anti-KSHV and anti-actin antibodies were either gifts from Ren Sun (University of California, Los Angeles, CA, USA) or purchased from Promab Inc (Richmond, CA, USA), Advanced Biotechnologies (Columbia, MD, USA), and Sigma (St. Louis, MO, USA).

### 4.2. Ribozyme Constructs and Assays

The dimethyl sulphate (DMS)-accessible regions [17,27,28] of RTA mRNA were mapped in accordance to procedures previously indicated [19,32]. BCBL-1 cells were first induced with TPA, then treated with 1% DMS for 5–10 min and washed with phosphate-buffered saline (PBS) supplemented with 1 mM β-mercaptoethanol. Total RNA was isolated and subjected to primer extension assays in the presence of [^32^P]-labeled primers hybridizing to various RTA mRNA regions [17,27,28]. The blocked primer extension sites representing the DMS-modified positions were revealed and analyzed with a Phosphorimager [19,32].

The DNA sequences for functional F-RTA and control C-RTA ribozymes were constructed by PCR from plasmid pFL117 with the M1 RNA coding sequence and plasmid pC102 with the mutant C102 coding sequence, correspondingly [29,30,47], using 5′ primer Rb-RTA5-AF25 (5′-GGAATTCTAATACGACTCACTATAG-3′) and 3′ primer Rb-RTA3 (5′-CCCGCTCGAGAAAAAATGGTGCATACGAAACAATCTACTGTGGAATTGTG-3′). We constructed the DNA sequence for the substrate rta-39 by PCR with 5′ primer rta-39-5-AF25 (5′-GGAATTCTAATACGACTCACTATAG-3′) and 3′ primer rta-39-3 (5′-CGGGATCCCGTAGATTGTTTCGTATGCGTCATTTATATCTATAGTGAGTCGTATTA-3′). We produced the ribozymes and RNA substrate rta-39 with in vitro transcription procedures [29,30,47].

Following previously described procedures, we assayed the ribozyme-mediated cleavage of rta-39 in vitro in buffer A (50 mM Tris, pH 7.5; 100 mM NH_4_Cl, 100 mM MgCl_2_) to obtain the overall cleavage rate (k_cat_/K_m_)^s^ [30,32,48]. We also measured the equilibrium dissociation constants (K_d_) of the complexes of the ribozymes and rta-39 in buffer E (50 mM Tris, pH 7.5; 100 mM NH_4_Cl, 100 mM CaCl_2_, 3% glycerol, 0.1% xylene cyanol, 0.1% bromophenol blue) [30,32,48]. The (k_cat_/K_m_)^s^ and K_d_ values were the means of three independent experiments.

### 4.3. Construction of Ribozyme-Expressing Cells

We cloned the sequence coding for F-RTA, C-RTA, and M1-TK into LXSN vector and under the control of the U6 RNA promoter to generate construct pLXSN-F-RTA, pLXSN-C-RTA, and pLXSN-M1-TK, respectively [17,29,31,49]. BCBL-1 cells were transfected with the constructs, selected with neomycin (600 µg/mL) (Invitrogen, Carlsbad, CA, USA), and cloned. Ribozyme expression was assessed by Northern blot analyses, following previously described procedures [17,29,31,49].

### 4.4. Assays of KSHV Gene Expression

We grew cells containing M1GSs both with and without TPA (20 ng/mL) for 4–48 h, and harvested RNA and protein samples from cells at different time points post-induction [26,34,46]. In Northern blot analyses, RNAs were separated in formaldehyde-agarose gels, transferred to membranes, hybridized with probes, and analyzed with a STORM 840 PhosphorImager [32,48]. In Western blot analyses, protein samples were separated on SDS-PAGE, transferred to membranes, reacted with antibodies, stained with a Western chemiluminescent substrate kit (Thermo Fisher, Waltham, MA, USA), and finally analyzed with a STORM 840 PhosphorImager [19,32]. We performed quantification with experiments in triplicate.

### 4.5. Assays of Latent Viral DNA Levels and Virus Growth

We performed qPCR to assess the KSHV lytic DNA level by amplifying the viral ORF25 sequence. We induced BCBL-1 (*n* = 1 × 10^6^) cells with TPA (20 ng/mL) for 24–48 h. Supernatants were collected from cultured cell media at different times post-induction and treated with DNase I (Promega, Madison, WI, USA). The DNase-resistant viral DNAs were extracted with phenol and precipitated with ethanol, and then subjected to qPCR with SYBR Green and 5′ primer ORF25-5 (5′-ACAGTTTATGGCACGCATAGTG-3′) and 3′ primer ORF25-3 (5′-GGTTCTCTGAATCTCGTCGTGT-3′) [50,51,52]. Values shown in the virus growth experiments represent the increased magnitudes of virion DNA level in the samples, as compared to the level of virion DNA found in BCBL-1 cells that did not contain a ribozyme and were incubated without TPA. We executed real-time qPCR in an iCycler (Bio-Rad, Hercules, CA, USA). The results were the averages of three independent experiments.

We performed qPCR to assess the KSHV latent DNA level by amplifying the viral ORF73 sequence. DNAs were purified from cells without TPA treatment and subsequently added to the qPCR mix containing SYBR Green, 5′ primer LANA5 (5′-GAGTCTGGTGACGACTTGGAG-3′) and 3′ primer LANA3 (5′-GAGTCTGGTGACGACTTGGAG-3′) [50,51,52]. We also assessed the actin DNA level by qPCR with 5′ primer Actin5 (5′-TGACGGGGTCACCCACACTGTGCCCATCTA-3′) and 3′ primer Actin3 (5′-CTAGAAGCATTGCGGTGGCAGATGGAGGG-3′) [53].

### 4.6. Statistical Analysis

Experiments were carried out in triplicate and repeated three times. Data were analyzed using statistical tools in GraphPad Prism software. We considered differences with *p* < 0.05 statistically significant.

## 5. Conclusions

In summary, our results show that an engineered ribozyme constructed from RNase P catalytic RNA can efficiently cleave an essential mRNA of Kaposi’s sarcoma-associated herpesvirus (KSHV) in vitro and effectively suppress the expression of its mRNA target in human cells. Moreover, expression of the constructed ribozyme in KSHV-infected cells caused substantial inhibition of viral replication and progeny production. Our results provide the first direct evidence of RNase P ribozyme having potential for anti-KSHV therapy.

## Figures and Tables

**Table 1:** Overall cleavage rate [(k_cat_/K_m_)^s^] and binding affinity (K_d_) in reactions of substrates rta-39 with RNase P ribozymes.

Enzyme	(k_cat_/K_m_)^s^ (µM^−1^·min^−1^)	K_d_ (nM)
F-RTA	0.27 ± 0.08	0.30 ± 0.07
C-RTA	<5 × 10^−6^	0.29 ± 0.06
M1-TK	<5 × 10^−6^	ND

The values are the averages derived from three independent experiments. “ND” not determined.

**Table 2:** Levels of suppression of viral gene expression in cells expressing ribozymes, compared to the levels of suppression in cells that were transfected with the LXSN empty vector and did not express a ribozyme (BCBL-1).

Viral Gene	Gene Class	BCBL-1	C-RTA	F-RTA	M1-TK
RTA mRNA	IE	0%	7%	94 ± 9%	0%
PAN RNA	Early	0%	5%	85 ± 7%	0%
ORF75 mRNA	Early/late	0%	2%	80 ± 6%	0%
RTA protein	IE	0%	5%	92 ± 7%	0%
ORF59 protein	Early	0%	4%	83 ± 6%	0%
ORF26 protein	Early/late	0%	2%	83 ± 6%	0%
K8.1 protein	Late	0%	0%	85 ± 8%	0%

The averages of three independent experiments are shown. Standard deviations of less than 5% are not shown.

## Data Availability

Not applicable.

## Supplementary Materials

The following supporting information can be downloaded at https://www.mdpi.com/article/10.3390/molecules28083619/s1. Table S1: The RTA mRNA regions modified by dimethyl sulphate (DMS).
