# Supplementary figures and images for: Suppressing Kaposi’s Sarcoma-Associated Herpesvirus Lytic Gene Expression and Replication by RNase P Ribozyme
Source: Molecules. 2023 Apr 21;28(8):3619. doi: 10.3390/molecules28083619 (PMC10142857; doi:10.3390/molecules28083619)

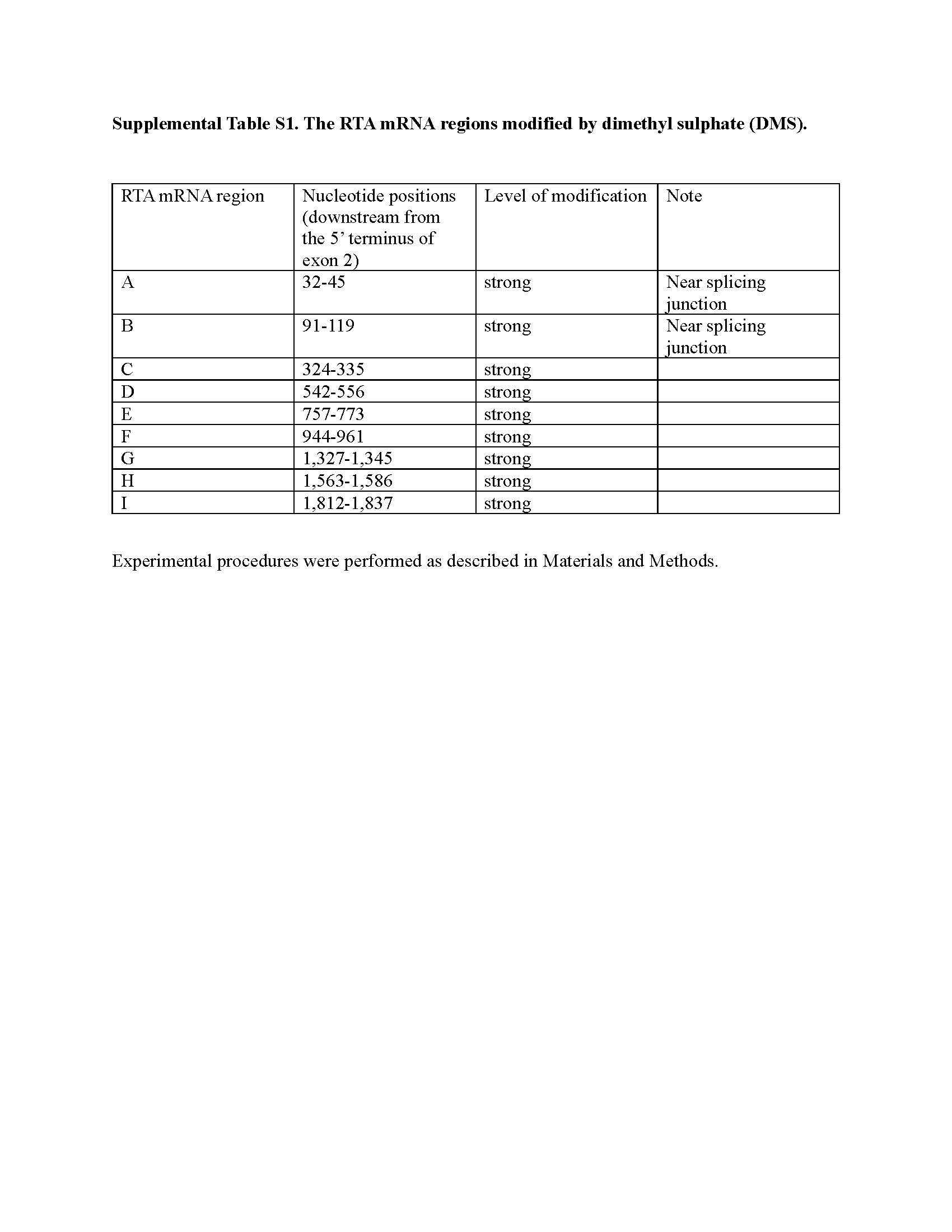

Supplement: Supplementary file 1 [file molecules-28-03619-s001.zip › molecules-2285456-supplementary.jpg]
